# Prospects for Observing and Localizing Gravitational-Wave Transients with Advanced LIGO and Advanced Virgo

**DOI:** 10.1007/lrr-2016-1

**Published:** 2016-02-08

**Authors:** B. P. Abbott, The LIGO Scientific Collaboration, Virgo Collaboration, R. Abbott, T. D. Abbott, M. R. Abernathy, F. Acernese, K. Ackley, C. Adams, T. Adams, P. Addesso, R. X. Adhikari, V. B. Adya, C. Affeldt, M. Agathos, K. Agatsuma, N. Aggarwal, O. D. Aguiar, A. Ain, P. Ajith, B. Allen, A. Allocca, P. A. Altin, D. V. Amariutei, S. B. Anderson, W. G. Anderson, K. Arai, M. C. Araya, C. C. Arceneaux, J. S. Areeda, N. Arnaud, K. G. Arun, G. Ashton, M. Ast, S. M. Aston, P. Astone, P. Aufmuth, C. Aulbert, S. Babak, P. T. Baker, F. Baldaccini, G. Ballardin, S. W. Ballmer, J. C. Barayoga, S. E. Barclay, B. C. Barish, D. Barker, F. Barone, B. Barr, L. Barsotti, M. Barsuglia, D. Barta, J. Bartlett, I. Bartos, R. Bassiri, A. Basti, J. C. Batch, C. Baune, V. Bavigadda, M. Bazzan, B. Behnke, M. Bejger, C. Belczynski, A. S. Bell, C. J. Bell, B. K. Berger, J. Bergman, G. Bergmann, C. P. L. Berry, D. Bersanetti, A. Bertolini, J. Betzwieser, S. Bhagwat, R. Bhandare, I. A. Bilenko, G. Billingsley, J. Birch, R. Birney, S. Biscans, A. Bisht, M. Bitossi, C. Biwer, M. A. Bizouard, J. K. Blackburn, C. D. Blair, D. Blair, R. M. Blair, S. Bloemen, O. Bock, T. P. Bodiya, M. Boer, G. Bogaert, C. Bogan, A. Bohe, P. Bojtos, C. Bond, F. Bondu, R. Bonnand, R. Bork, V. Boschi, S. Bose, A. Bozzi, C. Bradaschia, P. R. Brady, V. B. Braginsky, M. Branchesi, J. E. Brau, T. Briant, A. Brillet, M. Brinkmann, V. Brisson, P. Brockill, A. F. Brooks, D. A. Brown, D. D. Brown, N. M. Brown, C. C. Buchanan, A. Buikema, T. Bulik, H. J. Bulten, A. Buonanno, D. Buskulic, C. Buy, R. L. Byer, L. Cadonati, G. Cagnoli, C. Cahillane, J. Calderón Bustillo, T. Callister, E. Calloni, J. B. Camp, K. C. Cannon, J. Cao, C. D. Capano, E. Capocasa, F. Carbognani, S. Caride, J. Casanueva Diaz, C. Casentini, S. Caudill, M. Cavaglià, F. Cavalier, R. Cavalieri, G. Cella, C. Cepeda, L. Cerboni Baiardi, G. Cerretani, E. Cesarini, R. Chakraborty, T. Chalermsongsak, S. J. Chamberlin, M. Chan, S. Chao, P. Charlton, E. Chassande-Mottin, H. Y. Chen, Y. Chen, C. Cheng, A. Chincarini, A. Chiummo, H. S. Cho, M. Cho, J. H. Chow, N. Christensen, Q. Chu, S. Chua, S. Chung, G. Ciani, F. Clara, J. A. Clark, F. Cleva, E. Coccia, P.-F. Cohadon, A. Colla, C. G. Collette, M. Constancio, A. Conte, L. Conti, D. Cook, T. R. Corbitt, N. Cornish, A. Corsi, S. Cortese, C. A. Costa, M. W. Coughlin, S. B. Coughlin, J.-P. Coulon, S. T. Countryman, P. Couvares, D. M. Coward, M. J. Cowart, D. C. Coyne, R. Coyne, K. Craig, J. D. E. Creighton, J. Cripe, S. G. Crowder, A. Cumming, L. Cunningham, E. Cuoco, T. Dal Canton, S. L. Danilishin, S. D’Antonio, K. Danzmann, N. S. Darman, V. Dattilo, I. Dave, H. P. Daveloza, M. Davier, G. S. Davies, E. J. Daw, R. Day, D. DeBra, G. Debreczeni, J. Degallaix, M. De Laurentis, S. Deléglise, W. Del Pozzo, T. Denker, T. Dent, H. Dereli, V. Dergachev, R. DeRosa, R. De Rosa, R. DeSalvo, S. Dhurandhar, M. C. Díaz, L. Di Fiore, M. Di Giovanni, A. Di Lieto, I. Di Palma, A. Di Virgilio, G. Dojcinoski, V. Dolique, F. Donovan, K. L. Dooley, S. Doravari, R. Douglas, T. P. Downes, M. Drago, R. W. P. Drever, J. C. Driggers, Z. Du, M. Ducrot, S. E. Dwyer, T. B. Edo, M. C. Edwards, A. Effler, H.-B. Eggenstein, P. Ehrens, J. M. Eichholz, S. S. Eikenberry, W. Engels, R. C. Essick, T. Etzel, M. Evans, T. M. Evans, R. Everett, M. Factourovich, V. Fafone, H. Fair, S. Fairhurst, X. Fan, Q. Fang, S. Farinon, B. Farr, W. M. Farr, M. Favata, M. Fays, H. Fehrmann, M. M. Fejer, I. Ferrante, E. C. Ferreira, F. Ferrini, F. Fidecaro, I. Fiori, R. P. Fisher, R. Flaminio, M. Fletcher, J.-D. Fournier, S. Franco, S. Frasca, F. Frasconi, Z. Frei, A. Freise, R. Frey, T. T. Fricke, P. Fritschel, V. V. Frolov, P. Fulda, M. Fyffe, H. A. G. Gabbard, J. R. Gair, L. Gammaitoni, S. G. Gaonkar, F. Garufi, A. Gatto, G. Gaur, N. Gehrels, G. Gemme, B. Gendre, E. Genin, A. Gennai, J. George, L. Gergely, V. Germain, A. Ghosh, S. Ghosh, J. A. Giaime, K. D. Giardina, A. Giazotto, K. Gill, A. Glaefke, E. Goetz, R. Goetz, L. Gondan, G. González, J. M. Gonzalez Castro, A. Gopakumar, N. A. Gordon, M. L. Gorodetsky, S. E. Gossan, M. Gosselin, R. Gouaty, C. Graef, P. B. Graff, M. Granata, A. Grant, S. Gras, C. Gray, G. Greco, A. C. Green, P. Groot, H. Grote, S. Grunewald, G. M. Guidi, X. Guo, A. Gupta, M. K. Gupta, K. E. Gushwa, E. K. Gustafson, R. Gustafson, J. J. Hacker, B. R. Hall, E. D. Hall, G. Hammond, M. Haney, M. M. Hanke, J. Hanks, C. Hanna, M. D. Hannam, J. Hanson, T. Hardwick, J. Harms, G. M. Harry, I. W. Harry, M. J. Hart, M. T. Hartman, C.-J. Haster, K. Haughian, A. Heidmann, M. C. Heintze, H. Heitmann, P. Hello, G. Hemming, M. Hendry, I. S. Heng, J. Hennig, A. W. Heptonstall, M. Heurs, S. Hild, D. Hoak, K. A. Hodge, D. Hofman, S. E. Hollitt, K. Holt, D. E. Holz, P. Hopkins, D. J. Hosken, J. Hough, E. A. Houston, E. J. Howell, Y. M. Hu, S. Huang, E. A. Huerta, D. Huet, B. Hughey, S. Husa, S. H. Huttner, T. Huynh-Dinh, A. Idrisy, N. Indik, D. R. Ingram, R. Inta, H. N. Isa, J.-M. Isac, M. Isi, G. Islas, T. Isogai, B. R. Iyer, K. Izumi, T. Jacqmin, H. Jang, K. Jani, P. Jaranowski, S. Jawahar, F. Jiménez-Forteza, W. W. Johnson, D. I. Jones, R. Jones, R. J. G. Jonker, L. Ju, K. Haris, C. V. Kalaghatgi, V. Kalogera, S. Kandhasamy, G. Kang, J. B. Kanner, S. Karki, M. Kasprzack, E. Katsavounidis, W. Katzman, S. Kaufer, T. Kaur, K. Kawabe, F. Kawazoe, F. Kéfélian, M. S. Kehl, D. Keitel, D. B. Kelley, W. Kells, R. Kennedy, J. S. Key, A. Khalaidovski, F. Y. Khalili, S. Khan, Z. Khan, E. A. Khazanov, N. Kijbunchoo, C. Kim, J. Kim, K. Kim, N. Kim, Y.-M. Kim, E. J. King, P. J. King, D. L. Kinzel, J. S. Kissel, L. Kleybolte, S. Klimenko, S. M. Koehlenbeck, K. Kokeyama, S. Koley, V. Kondrashov, A. Kontos, M. Korobko, W. Z. Korth, I. Kowalska, D. B. Kozak, V. Kringel, B. Krishnan, A. Królak, C. Krueger, G. Kuehn, P. Kumar, L. Kuo, A. Kutynia, B. D. Lackey, M. Landry, J. Lange, B. Lantz, P. D. Lasky, A. Lazzarini, C. Lazzaro, P. Leaci, S. Leavey, E. Lebigot, C. H. Lee, H. K. Lee, H. M. Lee, K. Lee, A. Lenon, M. Leonardi, J. R. Leong, N. Leroy, N. Letendre, Y. Levin, B. M. Levine, T. G. F. Li, A. Libson, T. B. Littenberg, N. A. Lockerbie, J. Logue, A. L. Lombardi, J. E. Lord, M. Lorenzini, V. Loriette, M. Lormand, G. Losurdo, J. D. Lough, H. Lück, A. P. Lundgren, J. Luo, R. Lynch, Y. Ma, T. MacDonald, B. Machenschalk, M. MacInnis, D. M. Macleod, F. Magaña-Sandoval, R. M. Magee, M. Mageswaran, E. Majorana, I. Maksimovic, V. Malvezzi, N. Man, I. Mandel, V. Mandic, V. Mangano, G. L. Mansell, M. Manske, M. Mantovani, F. Marchesoni, F. Marion, S. Márka, Z. Márka, A. S. Markosyan, E. Maros, F. Martelli, L. Martellini, I. W. Martin, R. M. Martin, D. V. Martynov, J. N. Marx, K. Mason, A. Masserot, T. J. Massinger, M. Masso-Reid, F. Matichard, L. Matone, N. Mavalvala, N. Mazumder, G. Mazzolo, R. McCarthy, D. E. McClelland, S. McCormick, S. C. McGuire, G. McIntyre, J. McIver, D. J. McManus, S. T. McWilliams, D. Meacher, G. D. Meadors, J. Meidam, A. Melatos, G. Mendell, D. Mendoza-Gandara, R. A. Mercer, E. Merilh, M. Merzougui, S. Meshkov, C. Messenger, C. Messick, P. M. Meyers, F. Mezzani, H. Miao, C. Michel, H. Middleton, E. E. Mikhailov, L. Milano, J. Miller, M. Millhouse, Y. Minenkov, J. Ming, S. Mirshekari, C. Mishra, S. Mitra, V. P. Mitrofanov, G. Mitselmakher, R. Mittleman, A. Moggi, M. Mohan, S. R. P. Mohapatra, M. Montani, B. C. Moore, C. J. Moore, D. Moraru, G. Moreno, S. R. Morriss, K. Mossavi, B. Mours, C. M. Mow-Lowry, C. L. Mueller, G. Mueller, A. W. Muir, Arunava Mukherjee, D. Mukherjee, S. Mukherjee, A. Mullavey, J. Munch, D. J. Murphy, P. G. Murray, A. Mytidis, I. Nardecchia, L. Naticchioni, R. K. Nayak, V. Necula, K. Nedkova, G. Nelemans, M. Neri, A. Neunzert, G. Newton, T. T. Nguyen, A. B. Nielsen, S. Nissanke, A. Nitz, F. Nocera, D. Nolting, M. E. N. Normandin, L. K. Nuttall, J. Oberling, E. Ochsner, J. O’Dell, E. Oelker, G. H. Ogin, J. J. Oh, S. H. Oh, F. Ohme, M. Oliver, P. Oppermann, R. J. Oram, B. O’Reilly, R. O’Shaughnessy, C. D. Ott, D. J. Ottaway, R. S. Ottens, H. Overmier, B. J. Owen, A. Pai, S. A. Pai, J. R. Palamos, O. Palashov, C. Palomba, A. Pal-Singh, H. Pan, C. Pankow, F. Pannarale, B. C. Pant, F. Paoletti, A. Paoli, M. A. Papa, H. R. Paris, W. Parker, D. Pascucci, A. Pasqualetti, R. Passaquieti, D. Passuello, Z. Patrick, B. L. Pearlstone, M. Pedraza, R. Pedurand, L. Pekowsky, A. Pele, S. Penn, R. Pereira, A. Perreca, M. Phelps, O. Piccinni, M. Pichot, F. Piergiovanni, V. Pierro, G. Pillant, L. Pinard, I. M. Pinto, M. Pitkin, R. Poggiani, A. Post, J. Powell, J. Prasad, V. Predoi, S. S. Premachandra, T. Prestegard, L. R. Price, M. Prijatelj, M. Principe, S. Privitera, G. A. Prodi, L. Prokhorov, M. Punturo, P. Puppo, M. Pürrer, H. Qi, J. Qin, V. Quetschke, E. A. Quintero, R. Quitzow-James, F. J. Raab, D. S. Rabeling, H. Radkins, P. Raffai, S. Raja, M. Rakhmanov, P. Rapagnani, V. Raymond, M. Razzano, V. Re, J. Read, C. M. Reed, T. Regimbau, L. Rei, S. Reid, D. H. Reitze, H. Rew, F. Ricci, K. Riles, N. A. Robertson, R. Robie, F. Robinet, A. Rocchi, L. Rolland, J. G. Rollins, V. J. Roma, J. D. Romano, R. Romano, G. Romanov, J. H. Romie, D. Rosińska, S. Rowan, A. Rüdiger, P. Ruggi, K. Ryan, S. Sachdev, T. Sadecki, L. Sadeghian, M. Saleem, F. Salemi, A. Samajdar, L. Sammut, E. J. Sanchez, V. Sandberg, B. Sandeen, J. R. Sanders, B. Sassolas, B. S. Sathyaprakash, P. R. Saulson, O. Sauter, R. L. Savage, A. Sawadsky, P. Schale, R. Schilling, J. Schmidt, P. Schmidt, R. Schnabel, R. M. S. Schofield, A. Schönbeck, E. Schreiber, D. Schuette, B. F. Schutz, J. Scott, S. M. Scott, D. Sellers, D. Sentenac, V. Sequino, A. Sergeev, G. Serna, Y. Setyawati, A. Sevigny, D. A. Shaddock, S. Shah, M. S. Shahriar, M. Shaltev, Z. Shao, B. Shapiro, P. Shawhan, A. Sheperd, D. H. Shoemaker, D. M. Shoemaker, K. Siellez, X. Siemens, D. Sigg, A. D. Silva, D. Simakov, A. Singer, L. P. Singer, A. Singh, R. Singh, A. M. Sintes, B. J. J. Slagmolen, J. R. Smith, N. D. Smith, R. J. E. Smith, E. J. Son, B. Sorazu, F. Sorrentino, T. Souradeep, A. K. Srivastava, A. Staley, M. Steinke, J. Steinlechner, S. Steinlechner, D. Steinmeyer, B. C. Stephens, R. Stone, K. A. Strain, N. Straniero, G. Stratta, N. A. Strauss, S. Strigin, R. Sturani, A. L. Stuver, T. Z. Summerscales, L. Sun, P. J. Sutton, B. L. Swinkels, M. J. Szczepanczyk, M. Tacca, D. Talukder, D. B. Tanner, M. Tápai, S. P. Tarabrin, A. Taracchini, R. Taylor, T. Theeg, M. P. Thirugnanasambandam, E. G. Thomas, M. Thomas, P. Thomas, K. A. Thorne, K. S. Thorne, E. Thrane, S. Tiwari, V. Tiwari, K. V. Tokmakov, C. Tomlinson, M. Tonelli, C. V. Torres, C. I. Torrie, D. Töyrä, F. Travasso, G. Traylor, D. Trifirò, M. C. Tringali, L. Trozzo, M. Tse, M. Turconi, D. Tuyenbayev, D. Ugolini, C. S. Unnikrishnan, A. L. Urban, S. A. Usman, H. Vahlbruch, G. Vajente, G. Valdes, N. van Bakel, M. van Beuzekom, J. F. J. van den Brand, C. van den Broeck, D. C. Vander-Hyde, L. van der Schaaf, M. V. van der Sluys, J. V. van Heijningen, A. A. van Veggel, M. Vardaro, S. Vass, M. Vasúth, R. Vaulin, A. Vecchio, G. Vedovato, J. Veitch, P. J. Veitch, K. Venkateswara, D. Verkindt, F. Vetrano, A. Viceré, S. Vinciguerra, D. J. Vine, J.-Y. Vinet, S. Vitale, T. Vo, H. Vocca, C. Vorvick, W. D. Vousden, S. P. Vyatchanin, A. R. Wade, L. E. Wade, M. Wade, M. Walker, L. Wallace, S. Walsh, G. Wang, H. Wang, M. Wang, X. Wang, Y. Wang, R. L. Ward, J. Warner, M. Was, B. Weaver, L.-W. Wei, M. Weinert, A. J. Weinstein, R. Weiss, T. Welborn, L. Wen, P. Weßels, T. Westphal, K. Wette, J. T. Whelan, D. J. White, B. F. Whiting, R. D. Williams, A. R. Williamson, J. L. Willis, B. Willke, M. H. Wimmer, W. Winkler, C. C. Wipf, H. Wittel, G. Woan, J. Worden, J. L. Wright, G. Wu, J. Yablon, W. Yam, H. Yamamoto, C. C. Yancey, M. J. Yap, H. Yu, M. Yvert, A. Zadrożny, L. Zangrando, M. Zanolin, J.-P. Zendri, M. Zevin, F. Zhang, L. Zhang, M. Zhang, Y. Zhang, C. Zhao, M. Zhou, Z. Zhou, X. J. Zhu, M. E. Zucker, S. E. Zuraw, J. Zweizig

**Affiliations:** 1 LIGO, California Institute of Technology, Pasadena, CA 91125 USA; 2 Louisiana State University, Baton Rouge, LA 70803 USA; 3 Fisciano, Università di Salerno, I-84084 Salerno, Italy; 4 Sezione di Napoli, INFN, Complesso Universitario di Monte S.Angelo, I-80126 Napoli, Italy; 5 University of Florida, Gainesville, FL 32611 USA; 6 LIGO Livingston Observatory, Livingston, LA 70754 USA; 7 Laboratoire d’Annecy-le-Vieux de Physique des Particules (LAPP), Université Savoie Mont Blanc, CNRS/IN2P3, F-74941 Annecy-le-Vieux, France; 8 University of Sannio at Benevento, I-82100 Benevento, Italy; 9 Albert-Einstein-Institut, Max-Planck-Institut für Gravitationsphysik, D-30167 Hannover, Germany; 10 Nikhef, Science Park, 1098 XG Amsterdam, The Netherlands; 11 LIGO, Massachusetts Institute of Technology, Cambridge, MA 02139 USA; 12 Instituto Nacional de Pesquisas Espaciais, 12227-010 São José dos Campos, SP Brazil; 13 Inter-University Centre for Astronomy and Astrophysics, Pune, 411007 India; 14 International Centre for Theoretical Sciences, Tata Institute of Fundamental Research, Bangalore, 560012 India; 15 University of Wisconsin-Milwaukee, Milwaukee, WI 53201 USA; 16 Leibniz Universität Hannover, D-30167 Hannover, Germany; 17 Università di Pisa, I-56127 Pisa, Italy; 18 Sezione di Pisa, INFN, I-56127 Pisa, Italy; 19 Australian National University, Canberra, Australian Capital Territory 0200, Australia; 20 The University of Mississippi, University, MS 38677, Oxford, USA; 21 California State University Fullerton, Fullerton, CA 92831 USA; 22 LAL, Univ. Paris-Sud, CNRS/IN2P3, Université Paris-Saclay, Orsay, France; 23 Chennai Mathematical Institute, Chennai, India; 24 University of Southampton, Southampton, SO17 1BJ UK; 25 Universitäat Hamburg, D-22761 Hamburg, Germany; 26 Sezione di Roma, INFN, I-00185 Roma, Italy; 27 Albert-Einstein-Institut, Max-Planck-Institut für Gravitationsphysik, D-14476 Potsdam-Golm, Germany; 28 Montana State University, Bozeman, MT 59717 USA; 29 Università di Perugia, I-06123 Perugia, Italy; 30 Sezione di Perugia, INFN, I-06123 Perugia, Italy; 31 European Gravitational Observatory (EGO), I-56021 Cascina, Pisa, Italy; 32 Syracuse University, Syracuse, NY 13244 USA; 33 SUPA, University of Glasgow, Glasgow, G12 8QQ UK; 34 LIGO Hanford Observatory, Richland, WA 99352 USA; 35 APC, AstroParticule et Cosmologie, Université Paris Diderot, CNRS/IN2P3, CEA/Irfu, Observatoire de Paris, Sorbonne Paris Cité, F-75205 Paris Cedex 13, France; 36 RMKI, Wigner RCP, H-1121 Budapest, Konkoly Thege Miklós út 29-33, Hungary; 37 Columbia University, New York, NY 10027 USA; 38 Stanford University, Stanford, CA 94305 USA; 39 Dipartimento di Fisica e Astronomia, Università di Padova, I-35131 Padova, Italy; 40 Sezione di Padova, INFN, I-35131 Padova, Italy; 41 CAMK-PAN, 00-716 Warsaw, Poland; 42 Astronomical Observatory, Warsaw University, 00-478 Warsaw, Poland; 43 University of Birmingham, Birmingham, B15 2TT UK; 44 Università degli Studi di Genova, I-16146 Genova, Italy; 45 Sezione di Genova, INFN, I-16146 Genova, Italy; 46 RRCAT, Indore, MP, 452013 India; 47 Faculty of Physics, Lomonosov Moscow State University, Moscow, 119991 Russia; 48 SUPA, University of the West of Scotland, Paisley, PA1 2BE UK; 49 University of Western Australia, Crawley, Western Australia 6009 Australia; 50 Department of Astrophysics/IMAPP, Radboud University Nijmegen, P.O. Box 9010, 6500 GL Nijmegen, The Netherlands; 51 ARTEMIS, Université Côte d’Azur, CNRS and Observatoire de la Côte d’Azur, F-06304 Nice, France; 52 “Lendulet” Astrophysics Research Group, MTA Eötvös University, Budapest, 1117 Hungary; 53 Institut de Physique de Rennes, CNRS, Université de Rennes 1, F-35042 Rennes, France; 54 Washington State University, Pullman, WA 99164 USA; 55 Università degli Studi di Urbino ‘Carlo Bo’, I-61029 Urbino, Italy; 56 Sezione di Firenze, INFN, I-50019 Sesto Fiorentino, Firenze, Italy; 57 University of Oregon, Eugene, OR 97403 USA; 58 Laboratoire Kastler Brossel, UPMC-Sorbonne Universités, CNRS, ENS-PSL Research University, Collège de France, F-75005 Paris, France; 59 VU University Amsterdam, 1081 HV Amsterdam, The Netherlands; 60 University of Maryland, College Park, MD 20742 USA; 61 Center for Relativistic Astrophysics and School of Physics, Georgia Institute of Technology, Atlanta, GA 30332 USA; 62 Laboratoire des Matériaux Avancés (LMA), IN2P3/CNRS, Université de Lyon, F-69622 Villeurbanne, Lyon, France; 63 IAC3—IEEC, Universitat de les Illes Balears, E-07122 Palma de Mallorca, Spain; 64 Università di Napoli ‘Federico II’, Complesso Universitario di Monte S.Angelo, I-80126 Napoli, Italy; 65 NASA/Goddard Space Flight Center, Greenbelt, MD 20771 USA; 66 Canadian Institute for Theoretical Astrophysics, University of Toronto, Toronto, Ontario M5S 3H8 Canada; 67 Tsinghua University, Beijing, 100084 China; 68 University of Michigan, Ann Arbor, MI 48109 USA; 69 Università di Roma Tor Vergata, I-00133 Roma, Italy; 70 Sezione di Roma Tor Vergata, INFN, I-00133 Roma, Italy; 71 National Tsing Hua University, Hsinchu City, Taiwan, R.O.C.; 72 Charles Sturt University, Wagga Wagga, New South Wales 2678 Australia; 73 University of Chicago, Chicago, IL 60637 USA; 74 Caltech CaRT, Pasadena, CA 91125 USA; 75 Korea Institute of Science and Technology Information, Daejeon, 305-806 Korea; 76 Carleton College, Northfield, MN 55057 USA; 77 Gran Sasso Science Institute, INFN, I-67100 L’Aquila, Italy; 78 Université di Roma’ La Sapienza’, I-00185 Roma, Italy; 79 University of Brussels, Brussels, 1050 Belgium; 80 Texas Tech University, Lubbock, TX 79409 USA; 81 Cardiff University, Cardiff, CF24 3AA UK; 82 University of Minnesota, Minneapolis, MN 55455 USA; 83 The University of Melbourne, Parkville, Victoria 3010 Australia; 84 The University of Texas Rio Grande Valley, Brownsville, TX 78520 USA; 85 The University of Sheffield, Sheffield, S10 2TN UK; 86 Montclair State University, Montclair, NJ 07043 USA; 87 Dipartimento di Fisica, Università di Trento, I-38123 Povo, Trento, Italy; 88 Trento Institute for Fundamental Physics and Applications, INFN, I-38123 Povo, Trento, Italy; 89 The Pennsylvania State University, University Park, Pennsylvania, PA 16802 USA; 90 University of Cambridge, Cambridge, CB2 1TN UK; 91 Indian Institute of Technology, Gandhinagar, Ahmedabad Gujarat, 382424 India; 92 Institute for Plasma Research, Bhat, Gandhinagar, Ahmedabad, 382428 India; 93 University of Szeged, Dóm téer 9, Szeged, 6720 Hungary; 94 Embry-Riddle Aeronautical University, Prescott, AZ 86301 USA; 95 Tata Institute for Fundamental Research, Mumbai, 400005 India; 96 American University, Washington, D.C., 20016 USA; 97 University of Massachusetts-Amherst, Amherst, MA 01003 USA; 98 University of Adelaide, Adelaide, South Australia 5005 Australia; 99 West Virginia University, Morgantown, WV 26506 USA; 100 University of Bialystok, 15-424 Bialystok, Poland; 101 SUPA, University of Strathclyde, Glasgow, G1 1XQ UK; 102 IISER-TVM, CET Campus, Trivandrum Kerala, 695016 India; 103 Northwestern University, Evanston, IL 60208 USA; 104 Institute of Applied Physics, Nizhny Novgorod, 603950 Russia; 105 Pusan National University, Busan, 609-735 Korea; 106 Hanyang University, Seoul, 133-791 Korea; 107 NCBJ, 05-400 Świerk-Otwock, Poland; 108 IM-PAN, 00-956 Warsaw, Poland; 109 Rochester Institute of Technology, Rochester, NY 14623 USA; 110 Monash University, Clayton, Victoria 3800 Australia; 111 Seoul National University, Seoul, 151-742 Korea; 112 ESPCI, CNRS, F-75005 Paris, France; 113 Dipartimento di Fisica, Università di Camerino, I-62032 Camerino, Italy; 114 Southern University and A&M College, Baton Rouge, LA 70813 USA; 115 College of William and Mary, Williamsburg, VA 23187 USA; 116 Instituto de Física Teórica, University Estadual Paulista/ICTP South American Institute for Fundamental Research, São Paulo, SP 01140-070 Brazil; 117 IISER-Kolkata, Mohanpur, West Bengal, 741252 India; 118 HSIC, Chilton, Rutherford Appleton Laboratory, Didcot, Oxon OX11 0QX UK; 119 Whitman College, 280 Boyer Ave, Walla Walla, WA 9936 USA; 120 National Institute for Mathematical Sciences, Daejeon, 305-390 Korea; 121 Hobart and William Smith Colleges, Geneva, NY 14456 USA; 122 Institute of Astronomy, 65-265 Zielona Góra, Poland; 123 Andrews University, Berrien Springs, MI 49104 USA; 124 Università di Siena, I-53100 Siena, Italy; 125 Trinity University, San Antonio, TX 78212 USA; 126 University of Washington, Seattle, WA 98195 USA; 127 Abilene Christian University, Abilene, TX 79699 USA; 128 Sezione di Napoli, INFN, I-80100 Napoli, Italy

**Keywords:** Gravitational waves, Gravitational-wave detectors, Electromagnetic counterparts, Data analysis

## Abstract

We present a possible observing scenario for the Advanced LIGO and Advanced Virgo gravitational-wave detectors over the next decade, with the intention of providing information to the astronomy community to facilitate planning for multi-messenger astronomy with gravitational waves. We determine the expected sensitivity of the network to transient gravitational-wave signals, and study the capability of the network to determine the sky location of the source. We report our findings for gravitational-wave transients, with particular focus on gravitational-wave signals from the inspiral of binary neutron-star systems, which are considered the most promising for multi-messenger astronomy. The ability to localize the sources of the detected signals depends on the geographical distribution of the detectors and their relative sensitivity, and 90% credible regions can be as large as thousands of square degrees when only two sensitive detectors are operational. Determining the sky position of a significant fraction of detected signals to areas of 5 deg^2^ to 20 deg^2^ will require at least three detectors of sensitivity within a factor of ∼ 2 of each other and with a broad frequency bandwidth. Should the third LIGO detector be relocated to India as expected, a significant fraction of gravitational-wave signals will be localized to a few square degrees by gravitational-wave observations alone.

## Introduction

Advanced LIGO (aLIGO) [61, 9] and Advanced Virgo (AdV) [24, 23, 25] are kilometer-scale gravitational-wave (GW) detectors that are expected to yield direct observations of GWs. In this article we describe the currently projected schedule, sensitivity, and sky-localization accuracy for the GW-detector network. We discuss the proposed sequence of observing runs (designated O1, O2, O3, etc.) and the prospects for multi-messenger astronomy.

The purpose of this article is to provide information to the astronomy community to assist in the formulation of plans for the upcoming era of GW observations. In particular, we intend this article to provide the information required for assessing the features of programs for joint observation of GW events using electromagnetic, neutrino, or other facilities.

The full science of aLIGO and AdV is broad [8], and is not covered in this article. We concentrate solely on candidate GW transient signals. We place particular emphasis on the coalescence of binary neutron-star (BNS) systems, which are the GW source for which electromagnetic follow-up seems most promising. For more general introductory articles on GW generation, detection and astrophysics, we point readers to [33, 87, 94].

Although our collaborations have amassed a great deal of experience with GW detectors and analysis, it is still difficult to make predictions for both improvements in search methods and for the rate of progress for detectors which are not yet fully installed or operational. *The scenarios of LIGO and Virgo detector sensitivity evolution and observing times given here represent our best estimates as of January 2016. They should not be considered as fixed or firm commitments*.

As the detectors’ construction and commissioning progress, we intend to release updated versions of this article. This is the second version of the article, written to coincide with the first observing run (O1) of the advanced-detector era. Changes with respect to the first version [4] are given in Appendix A. Progress has been made in the commissioning of the detectors, and the plausible observing scenarios are largely the same; the predicted sky-localization accuracies have been updated following improvements in parameter estimation.

## Commissioning and Observing Phases

We divide the development of the aLIGO and AdV observatories into three components:

- **Construction** includes the installation and testing of the detectors. This phase ends with *acceptance* of the detectors. Acceptance means that the interferometers can lock for periods of hours: light is resonant in the arms of the interferometer with no *guaranteed GW sensitivity*. Construction incorporates several short *engineering runs* with no astrophysical output as the detectors progress towards acceptance. The aLIGO construction project ended (on time and on budget) in March 2015. The acceptance of AdV is expected in the first part of 2016.
- **Commissioning** takes the detectors from their configuration at acceptance through progressively better sensitivity to the design advanced-generation detector sensitivity. Engineering runs in the commissioning phase allow us to understand our detectors and analyses in an observational mode; these are not intended to produce astrophysical results, but that does not preclude the possibility of this happening. Rather than proceeding directly to design sensitivity before making astrophysical observations, commissioning is interleaved with *observing runs* of progressively better sensitivity.
- **Observing** runs begin when the detectors have reached (and can stably maintain) a significantly improved sensitivity compared with previous operation. It is expected that observing runs will produce astrophysical results, including upper limits on the rate of sources and possibly the first detections of GWs. During this phase, exchange of GW candidates with partners outside the LIGO Scientific Collaboration (LSC) and the Virgo Collaboration will be governed by memoranda of understanding (MOUs) [17, 2]. After the first four detections, we expect free exchange of GW event candidates with the astronomical community and the maturation of GW astronomy.

The progress in sensitivity as a function of time will affect the duration of the runs that we plan at any stage, as we strive to minimize the time to successful GW observations. Commissioning is a complex process which involves both scheduled improvements to the detectors and tackling unexpected new problems. While our experience makes us cautiously optimistic regarding the schedule for the advanced detectors, we are targeting an order of magnitude improvement in sensitivity relative to the previous generation of detectors over a wider frequency band. Consequently, it is not possible to make concrete predictions for sensitivity or duty cycle as a function of time. We can, however, use our experience as a guide to plausible scenarios for the detector operational states that will allow us to reach the desired sensitivity. Unexpected problems could slow down the commissioning, but there is also the possibility that progress may happen faster than predicted here. As the detectors begin to be commissioned, information on the cost in time and benefit in sensitivity will become more apparent and drive the schedule of runs. More information on event rates, including the first detection, could also change the schedule and duration of runs.

In Section 2.1 we present the commissioning plans for the aLIGO and AdV detectors. A summary of expected observing runs is in Section 2.2.

### Commissioning and observing roadmap

**Figure 1:**
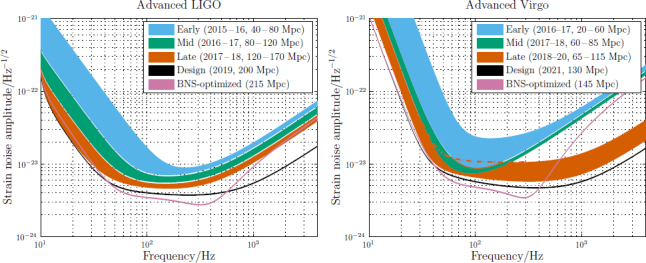
aLIGO (*left*) and AdV (*right*) target strain sensitivity as a function of frequency. The binary neutron-star (BNS) range, the average distance to which these signals could be detected, is given in megaparsec. Current notions of the progression of sensitivity are given for early, mid and late commissioning phases, as well as the final design sensitivity target and the BNS-optimized sensitivity. While both dates and sensitivity curves are subject to change, the overall progression represents our best current estimates.

The anticipated strain sensitivity evolution for aLIGO and AdV is shown in Figure 1. A standard figure of merit for the sensitivity of an interferometer is the BNS *range R*_BNS_: the volume- and orientation-averaged distance at which a compact binary coalescence consisting of two 1.4 *M*_⊙_ neutron stars gives a matched filter signal-to-noise ratio (SNR) of 8 in a sing le detector [58].^1^ The BNS ranges for the various stages of aLIGO and AdV expected evolution are also provided in Figure 1.

The commissioning of aLIGO is well under way. The original plan called for three identical 4-km interferometers, two at Hanford (H1 and H2) and one at Livingston (L1). In 2011, the LIGO Lab and IndIGO consortium in India proposed installing one of the aLIGO Hanford detectors (H2) at a new observatory in India (LIGO-India) [64]. As of early 2015, LIGO Laboratory has placed the H2 interferometer in long-term storage for possible use in India. Funding for the Indian portion of LIGO-India is in the final stages of consideration by the Indian government.

Advanced LIGO detectors began taking sensitive data in August 2015 in preparation for the first observing run. O1 formally began 18 September 2015 and ended 12 January 2016. It involved the H1 and L1 detectors; the detectors were not at full design sensitivity. We aimed for a BNS range of 40–80 Mpc for both instruments (see Figure 1), and both instruments were running with a 60–80 Mpc range. Subsequent observing runs will have increasing duration and sensitivity. We aim for a BNS range of 80–170 Mpc over 2016–2018, with observing runs of several months. Assuming that no unexpected obstacles are encountered, the aLIGO detectors are expected to achieve a 200 Mpc BNS range circa 2019. After the first observing runs, circa 2020, it might be desirable to optimize the detector sensitivity for a specific class of astrophysical signals, such as BNSs. The BNS range may then become 215 Mpc. The sensitivity for each of these stages is shown in Figure 1.

As a consequence of the planning for the installation of one of the LIGO detectors in India, the installation of the H2 detector has been deferred. This detector will be reconfigured to be identical to H1 and L1 and will be installed in India once the LIGO-India Observatory is complete. The final schedule will be adopted once final funding approvals are granted. If project approval comes soon, site development could start in 2016, with installation of the detector beginning in 2020. Following this scenario, the first observing runs could come circa 2022, and design sensitivity at the same level as the H1 and L1 detectors is anticipated for no earlier than 2024.

The time-line for the AdV interferometer (V1) [23] is still being defined, but it is anticipated that in 2016 AdV will join the aLIGO detectors in their second observing run (O2). Following an early step with sensitivity corresponding to a BNS range of 20–60 Mpc, commissioning is expected to bring AdV to a 60–85 Mpc in 2017–2018. A configuration upgrade at this point will allow the range to increase to approximately 65–115 Mpc in 2018–2020. The final design sensitivity, with a BNS range of 130 Mpc, is anticipated circa 2021. The corresponding BNS-optimized range would be 145 Mpc. The sensitivity curves for the various AdV configurations are shown in Figure 1.

The GEO 600 [76] detector will likely be operational in the early to middle phase of the AdV and aLIGO observing runs, i.e. 2015–2017. The sensitivity that potentially can be achieved by GEO in this time-frame is similar to the AdV sensitivity of the early and mid scenarios at frequencies around 1 kHz and above. GEO could therefore contribute to the detection and localization of high-frequency transients in this period. However, in the ∼ 100 Hz region most important for BNS signals, GEO will be at least 10 times less sensitive than the early AdV and aLIGO detectors, and will not contribute significantly.

Japan has begun the construction of an advanced detector, KAGRA [100, 28]. KAGRA is designed to have a BNS range comparable to AdV at final sensitivity. We do not consider KAGRA in this article, but the addition of KAGRA to the worldwide GW-detector network will improve both sky coverage and localization capabilities beyond those envisioned here [96].

Finally, further upgrades to the LIGO and Virgo detectors, within their existing facilities (e.g., [63, 78, 11]) as well as future underground detectors (for example, the Einstein Telescope [93]) are envisioned in the future. These affect both the rates of observed signals as well as the localizations of these events, but this lies beyond the scope of this paper.

### Envisioned observing schedule

Keeping in mind the important caveats about commissioning affecting the scheduling and length of observing runs, the following is a plausible scenario for the operation of the LIGO-Virgo network over the next decade:

- **2015–2016 (O1)** A four-month run (beginning 18 September 2015 and ending 12 January 2016) with the two-detector H1L1 network at early aLIGO sensitivity (40–80 Mpc BNS range).
- **2016–2017 (O2)** A six-month run with H1L1 at 80–120 Mpc and V1 at 20–60 Mpc.
- **2017–2018 (O3)** A nine-month run with H1L1 at 120–170 Mpc and V1 at 60–85 Mpc.
- **2019+** Three-detector network with H1L1 at full sensitivity of 200 Mpc and V1 at 65–115 Mpc.
- **2022+** H1L1V1 network at full sensitivity (aLIGO at 200 Mpc, AdV at 130 Mpc), with other detectors potentially joining the network. Including a fourth detector improves sky localization [72, 109, 79, 91], so as an illustration we consider adding LIGO-India to the network. 2022 is the earliest time we imagine LIGO-India could be operational, and it would take several more years for it to achieve full sensitivity.

**Figure 2:**
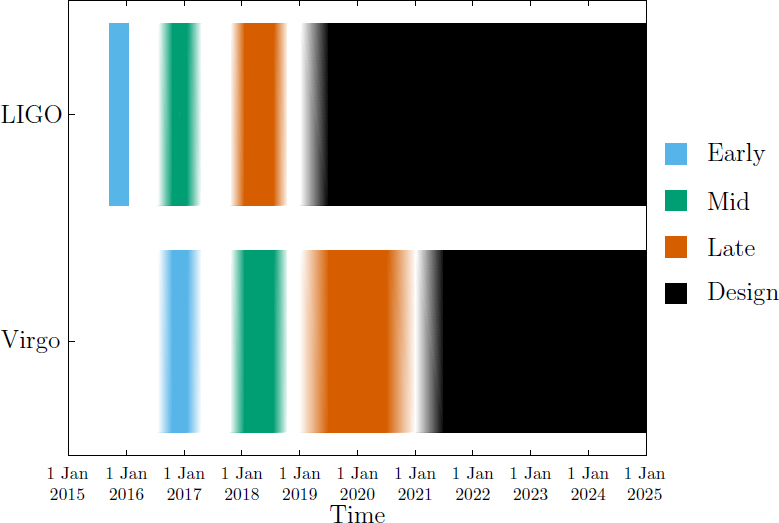
The planned sensitivity evolution and observing runs of the aLIGO and AdV detectors over the coming years. The colored bars show the observing runs, with the expected sensitivities given by the data in Figure 1. There is significant uncertainty in the start and end times of the observing runs, especially for those further in the future, and these could move forward or backwards by a few months relative to what is shown above. The plan is summarised in Section 2.2.

This time-line is summarized in Figure 2. The observational implications of this scenario are discussed in Section 4.

## Searches for Gravitational-Wave Transients

Data from GW detectors are searched for many types of possible signals [8]. Here we focus on signals from compact binary coalescences (CBCs), including BNS systems, and on generic transient or *burst* signals. See [19, 18, 14] for observational results from LIGO and Virgo for such systems.

The rate of BNS coalescences is uncertain [54]. For this work we adopt the estimates of [13], which predicts the rate to lie between 10^−8^–10^−5^ Mpc^−3^ yr^−1^, with a most plausible value of 10^−6^ Mpc^−3^ yr^−1^; this corresponds to 0.4–400 signals above an SNR of 8 per year of observation for a single aLIGO detector at final sensitivity, and a best estimate of 40 BNS signals per year [13]. Rate estimation remains an active area of research (e.g., [69, 50, 49, 47]), and will be informed by the number of detections (or lack thereof) in observing runs.

While the intrinsic rates of neutron star-black hole (NS-BH) and binary black hole (BBH) mergers are expected to be a factor of tens or hundreds lower than the BNS rate, the distance to which they can be observed is a factor of two to five larger. Consequently, the predicted observable rates are similar [13, 92]. Expected rates for other transient sources are lower and/or less well constrained.

The gravitational waveform from a BNS coalescence is well modeled and matched filtering can be used to search for signals and measure the system parameters [74, 35, 34, 90]. For systems containing black holes, or in which the component spin is significant, uncertainties in the waveform model can reduce the sensitivity of the search [81, 62, 45, 103, 85, 95, 68]. Searches for bursts make few assumptions on the signal morphology, using time-frequency decompositions to identify statistically significant excess-power transients in the data. Burst searches generally perform best for short-duration signals (≲ 1 s), although search development remains an area of active research (e.g., [71, 102, 40, 106, 26, 104, 43, 105, 66]); their astrophysical targets include core-collapse supernovae, magnetar flares, BBH coalescences, cosmic string cusps, and, possibly, as-yet-unknown systems.

In the era of advanced detectors, the LSC and Virgo will search in *near real-time* for CBC and burst signals for the purpose of rapidly identifying event candidates. A prompt notice of a potential GW transient by LIGO-Virgo might enable follow-up observations in the electromagnetic spectrum. A first follow-up program including low-latency analysis, event candidate selection, position reconstruction and the sending of alerts to several observing partners (optical, X-ray, and radio) was implemented and exercised during the 2009–2010 LIGO-Virgo science run [16, 15, 53]. Latencies of less than 1 hour were achieved and we expect to improve this in the advanced-detector era. Increased detection confidence, improved sky localization, and identification of host galaxy and redshift are just some of the benefits of joint GW-electromagnetic observations. With this in mind, we focus on two points of particular relevance for follow-up of GW events: the source localization afforded by a GW network as well as the relationship between signal significance, or false alarm rate (FAR), and source localization.

### Detection and false alarm rates

The rate of false alarm triggers above a given SNR depends critically upon the data quality of the advanced detectors; non-stationary transients or *glitches* [1, 10] produce an elevated background of loud triggers. For low-mass binary coalescence searches, the waveforms are well modeled, and signal consistency tests reduce the background significantly [27, 38, 107]. For burst sources which are not well modeled, or which spend only a short time in the detectors’ sensitive band, it is more difficult to distinguish between the signal and a glitch, and so a reduction of the FAR comes at a higher cost in terms of reduced detection efficiency.

**Figure 3:**
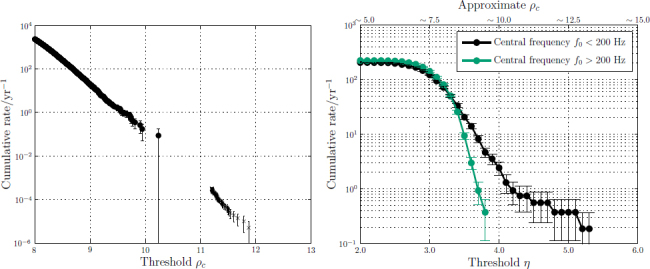
False alarm rate versus detection statistic for compact binary coalescence (CBC) and burst searches on 2009–2010 LIGO-Virgo data. *Left*: Cumulative rate of background events for a subset of the CBC search parameter space, as a function of the threshold ranking statistic *ρ*_*c*_ [19]. Different methods were used to estimate the background for rate for high and low *ρ*_*c*_, which is why there is an apparent gap in the data points. The background for the full search was approximately a factor of six higher. *Right*: Cumulative rate of background events for the burst search, as a function of the coherent network amplitude *η* [14]. For ease of comparison, we have also plotted the approximate equivalent *ρ*_*c*_ for the burst search (an exact identification is not possible as the search methods differ). The burst events are divided into two sets based on their central frequency.

Figure 3 shows the noise background as a function of detection statistic for the low-mass binary coalescence and burst searches with the 2009–2010 LIGO-Virgo data [19, 14].^2^ For binary mergers, the background rate decreases by a factor of ∼ 100 for every unit increase in combined SNR *ρ*_*c*_. Here *ρ*_*c*_ is a combined, re-weighted SNR [29, 19]. The re-weighting is designed to reduce the SNR of glitches while leaving signals largely unaffected. Consequently, for a signal, *ρ*_*c*_ is essentially the root-sum-square of the SNRs in the individual detectors. For bursts, we use the coherent network amplitude *η*, which measures the degree of correlation between the detectors [22, 86]. Glitches have little correlated energy and so give low values of *η*. Both *ρ*_*c*_ and *η* give an indication of the amplitude of the signal and can be used to rank events.

For CBC signals, we conservatively estimate that a *ρ*_*c*_ threshold of 12 is required for a FAR below ∼ 10^−2^ yr^−1^ in aLIGO-AdV. To arrive at this estimate, we begin with Figure 3 which indicates that an SNR of around 10.5 corresponds to a FAR of 10^−2^ yr^−1^. However, this corresponds to only a subspace of the CBC parameter space and the background of the full search is a factor of six higher. Additionally, due to the improved low frequency sensitivity of the advanced detectors and the inclusion of templates for binaries with (aligned) component spins, at least ten times as many waveform templates are required to perform the search [84, 34, 88, 46]. The background increases approximately linearly with the number of templates required. Consequently, we expect a background around a factor of 100 higher than indicated by Figure 3, which leads us to quote a threshold of 12 for a FAR of 10^−2^ yr^−1^ [31]. A combined SNR of 12 corresponds to a single-detector SNR of 8.5 in each of two detectors, or 7 in each of three detectors.

Instrumental disturbances in the data can have a greater effect on the burst search. At frequencies above 200 Hz, the rate of background events falls off steeply as a function of amplitude. At lower frequencies, however, the data often exhibit a significant tail of loud background events that are not simply removed by multi-detector consistency tests. Although the advanced detectors are designed with many technical improvements, we anticipate that similar features will persist, particularly at low frequencies. Improvements to detection pipelines, which better distinguish between glitches and potential waveforms, can help eliminate these tails (e.g., [66]). For a given FAR, the detection threshold may need to be tuned for different frequency ranges; for the initial detectors, a threshold of *η* ≳ 4.5–6 (approximately equivalent to *ρ*_*c*_ ≳ 12 from Figure 3) was needed for a FAR of 1/8 yr^−1^ [14]. The unambiguous observation of an electromagnetic counterpart could increase the detection confidence.

### Sky localization

Following the detection by the aLIGO-AdV network of a GW transient, determining the source’s location is a question for parameter estimation. Typically, posterior probability distributions for the sky position are constructed following a Bayesian framework [110, 43, 98]. Information comes from the time of arrival, plus the phase and amplitude of the GW.

An intuitive understanding of localization can be gained by considering triangulation using the observed time delays between sites [55, 56]. The effective single-site timing accuracy is approximately

1 $${\sigma _t} = {1 \over {2\pi \,\rho {\sigma _f}}},$$

where *ρ* is the SNR in the given detector and *σ*_*f*_ is the effective bandwidth of the signal in the detector, typically of order 100 Hz. Thus a typical timing accuracy is on the order of 10^−4^ s (about 1/100 of the light travel time between sites). This sets the localization scale. The simple model of equation (1) ignores many other relevant issues such as information from the signal amplitudes across the detector network, uncertainty in the emitted gravitational waveform, instrumental calibration accuracies, and correlation of sky location with other binary parameters [55, 112, 111, 80, 109, 79, 99, 31, 98]. While many of these affect the measurement of the time of arrival in individual detectors, such factors are largely common between two similar detectors, so the time difference between the two detectors is relatively uncorrelated with these nuisance parameters.

The triangulation approach underestimates how well a source can be localized, since it does not include all the relevant information. Its predictions can be improved by introducing the requirement of phase consistency between detectors [60]. Triangulation always performs poorly for a two-detector network, but, with the inclusion of phase coherence, can provide an estimate for the average performance of a three-detector network [31].^3^

**Figure 4:**
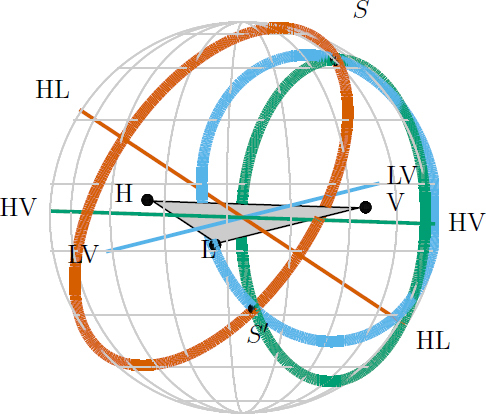
Source localization by triangulation for the aLIGO-AdV network. The locations of the three detectors are indicated by black dots, with LIGO Hanford labeled H; LIGO Livingston as L, and Virgo as V. The locus of constant time delay (with associated timing uncertainty) between two detectors forms an annulus on the sky concentric about the baseline between the two sites (labeled by the two detectors). For three detectors, these annuli may intersect in two locations. One is centered on the true source direction (*S*), while the other (*S*′) is its mirror image with respect to the geometrical plane passing through the three sites. For four or more detectors there is a unique intersection region of all of the annuli. Figure adapted from [41].

**Figure 5:**
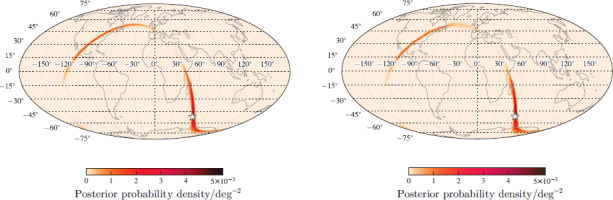
Posterior probability density for sky location for an example binary neutron-star coalescence observed with a two-detector network. *Left*: Map produced by the low-latency BAYESTAR code [99, 98]. *Right*: Map produced by the higher-latency (non-spinning) LALInference [110], which also produces posterior estimates for other parameters. These algorithms are discussed in Section 3.2.1. The star indicates the true source location. The event has a network signal-to-noise ratio of *ρ*_*c*_ = 13.2 using a noise curve appropriate for the first aLIGO run (O1, see Section 4.1). The plot is a Mollweide projection in geographic coordinates. Image reproduced with permission from [31], copyright by APS; further mock sky maps for the first two observing runs can be found at www.ligo.org/scientists/first2years/ for binary neutron-star signals and www.ligo.org/scientists/burst-first2years/ for burst signals.

Source localization using only timing for a two-site network yields an annulus on the sky; see Figure 4. Additional information such as signal amplitude, spin, and precession effects resolve this to only parts of the annulus, but even then sources will only be localized to regions of hundreds to thousands of square degrees [99, 31]. An example of a two-detector BNS localization is shown in Figure 5. The posterior probability distribution is primarily distributed along a ring, but this ring is broken, such that there are clear maxima.

For three detectors, the time delays restrict the source to two sky regions which are mirror images with respect to the plane passing through the three sites. It is often possible to eliminate one of these regions by requiring consistent amplitudes in all detectors. For signals just above the detection threshold, this typically yields regions with areas of several tens to hundreds of square degrees. Additionally, for BNSs, it is often possible to obtain a reasonable estimate of the distance to the source [109, 31], which can be used to further aid electromagnetic observations [79, 32]. If there is significant difference in sensitivity between detectors, the source is less well localized and we may be left with the majority of the annulus on the sky determined by the two most sensitive detectors. With four or more detectors, timing information alone is sufficient to localize to a single sky region, and the additional baselines help to limit the region to under 10 deg^2^ for some signals.

From Eq. (1), it follows that the *linear* size of the localization ellipse scales inversely with the SNR of the signal and the frequency bandwidth of the signal in the detector [31]. For GWs that sweep across the band of the detector, such as CBC signals, the effective bandwidth is ∼ 100 Hz, determined by the most sensitive frequencies of the detector. For shorter transients the bandwidth *ρ*_*f*_ depends on the specific signal. For example, GWs emitted by various processes in core-collapse supernovae are anticipated to have relatively large bandwidths, between 150–500 Hz [48, 82, 113, 83], largely independent of detector configuration. By contrast, the sky localization region for narrowband burst signals may consist of multiple disconnected regions and exhibit fringing features; see for example [72, 16, 51].

Some GW searches are triggered by electromagnetic observations, and in these cases localization information is known *a priori*. For example, in GW searches triggered by gamma-ray bursts [18, 7, 6] the triggering space-based telescope provides the localization. The detection of a GW transient associated with a gamma-ray burst would provide strong evidence for the burst progenitor; for example, BNS mergers are considered the likely progenitors of most short gamma-ray bursts. It is therefore important to have high-energy telescopes operating during the advanced-detector era. Furthermore, the rapid identification of a GW counterpart to such a trigger could prompt further spectroscopic studies and longer, deeper follow-up in different wavelengths that may not always be done in response to gamma-ray bursts. This is particularly important for gamma-ray bursts with larger sky localization uncertainties, such as those reported by the Fermi GBM, which are not followed up as frequently as bursts reported by Swift.

Finally, all GW data are stored permanently, so that it is possible to perform retroactive analyses at any time.

#### Localization of binary neutron stars

Providing prompt localizations for GW signals helps to maximise the chance that electromagnetic observatories can catch a counterpart. Sky maps will be produced at several different latencies, with updates coming from more computationally expensive algorithms that refine our understanding of the source. For BNS signals, rapid sky localization is performed using BAYESTAR [98], a Bayesian parameter-estimation code that computes source location using output from the detection pipeline. It can produce sky maps (as in Figure 5) with latencies of only a few seconds. A similar approach to low-latency localization has been separately developed by [42], who find results consistent with bayestar.

At higher latency, CBC parameter estimation is performed using the stochastic sampling algorithms of LALInference [110]. LALInference constructs posterior probability distributions for system parameters (not just location, but also mass, orientation, etc. [3]) by matching GW templates to the detector strain [44, 65]. Computing these waveforms is computationally expensive; this expense increases as the detectors’ low-frequency sensitivity improves and waveforms must be computed down to lower frequencies. The quickest LALInference BNS follow-up is computed using waveforms that do not include the full effects of component spin [99, 31], sky locations can then be reported with latency of hours to a couple of days. Parameter estimation is then performed using more accurate waveform approximates (those that include the full effects of spin precession), which can take weeks or months [57]. Methods of reducing the computational cost are actively being investigated (e.g., [37, 89, 36]).

There is negligible difference between the low-latency bayestar and the mid-latency non-spinning LALInference analyses if the BNS signal is loud enough to produce a trigger in all detectors: if there is not, LALInference currently gives a more precise localization, as it still uses information from the non-triggered detector [99, 98]. It is hoped to improve bayestar localization in the future by including information about sub-threshold signals. Provided that BNSs are slowly spinning [77], there should be negligible difference between the mid-latency non-spinning LALInference and the high-latency fully spinning LALInference analyses [57].

**Figure 6:**
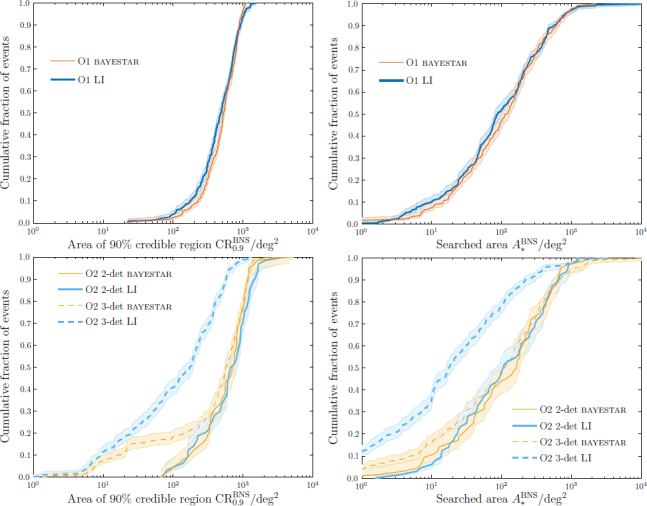
Anticipated binary neutron-star sky localization during the first two observing runs (*top*: O1, see Section 4.1; *bottom*: O2, see Section 4.2). The plots show the cumulative fractions of events with sky-localization areas smaller than the abscissa value. *Left*: Sky area of 90% credible region ${\rm{CR}}_{0.9}^{{\rm{BNS}}}$, the (smallest) area enclosing 90% of the total posterior probability. *Right*: Searched area $A_{\ast}^{{\rm{BNS}}}$, the area of the smallest credible region containing the true position. Results are shown for the low-latency bayestar [98] and higher-latency (non-spinning) LALInference (LI) [110] codes. The O2 results are divided into those where two detectors (2-det) are operating in coincidence, and those where three detectors (3-det) are operating: assuming a duty cycle of ∼ 80% for each instrument, the two-detector network would be operating for ∼ 40% of the total time and the three-detector network ∼ 50% of the time. The shaded areas indicate the 68% confidence intervals on the cumulative distributions. A detection threshold of a signal-to-noise ratio of 12 is used and results are taken from [31, 99].

Results from an astrophysically motivated population of BNS signals, assuming a detection threshold of a SNR of 12, are shown in Figure 6 [99, 31]. Sky localization is measured by the 90% credible region CR_0.9_, the smallest area enclosing 90% of the total posterior probability, and the searched area A_*_, the area of the smallest credible region that encompasses the true position [97]: CR_0.9_ gives the area of the sky that must be covered to expect a 90% chance of including the source location, and A_*_ gives the area that would be viewed before the true location using the given sky map. Results from both the low-latency bayestar and mid-latency non-spinning LALInference analyses are shown. These are discussed further in Section 4.1 and Section 4.2. The two-detector localizations get slightly worse going from O1 to O2. This is because although the detectors improve in sensitivity at every frequency, with the assumed noise curves the BNS signal bandwidth is lower in O2 for a given SNR because of enhanced sensitivity at low frequencies [99]. Despite this, the overall sky-localization in O2 is better than in O1. As sky localization improves with the development of the detector network [96, 72, 109, 91], these results mark the baseline of performance for the advanced-detector era.

#### Localization of bursts

The lowest latency burst sky maps are produced as part of the coherent WaveBurst (cWB) [71, 73] detection pipeline, earlier versions of which were used in prior burst searches [12, 14, 70]. Sky maps are produced using a constrained likelihood algorithm that coherently combines data from all the detectors; unlike other approaches, this is not fully Bayesian. The resulting sky maps currently show calibration issues.^4^ The confidence is over-estimated at low levels and under-estimated at high levels. However, the searched areas are consistent with those from other burst techniques, indicating that cWB maps can be used to guide observations. The cWB sky maps calculated with a latency of a few minutes; following detection, further parameter-estimation codes analyze the data.

At higher latency, burst signals are analyzed by LALInferenceBurst (LIB), a stochastic sampling algorithm similar to the LALInference code used to reconstruct CBC signals [110], and BayesWave, a reversible jump Markov-chain Monte Carlo algorithm that models both signals and glitches [43]. LIB uses a sine-Gaussian waveform (in place of the CBC templates used by LALInference), and can produce sky maps in a few hours. BayesWave uses a variable number of sine-Gaussian wavelets to model a signal and glitches while also fitting for the noise spectrum using BayesLine [75]; it produces sky maps with a latency of a few days. Sky maps produced by LIB and BayesWave should be similar, with performance depending upon the actual waveform.

**Figure 7:**
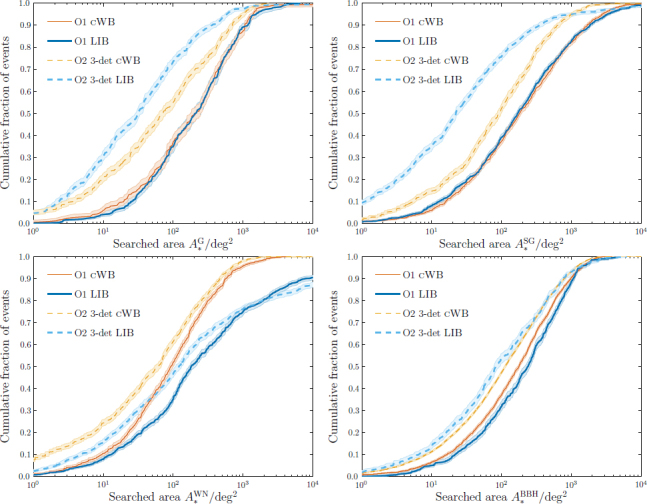
Simulated sky localization for Gaussian (G; *top left*), sine-Gaussian (SG; *top right*), broadband white-noise (WN; *bottom left*) and binary black-hole (BBH; *bottom right*) bursts during the first two observing runs (O1, see Section 4.1, and O2, see Section 4.2). The plots show the cumulative fractions of events with searched areas A_*_ smaller than the abscissa value. Results are shown for the low-latency coherent WaveBurst (cWB) [70, 71, 73] and higher-latency LALInferenceBurst (LIB) [110] codes. The O2 results consider only a three-detector (3-det) network; assuming an instrument duty cycle of ∼ 80%, this would be operational ∼ 50% of the time. The shaded areas indicate the 68% confidence intervals on the cumulative distributions. A detection threshold of a false alarm rate of approximately 1 yr^−1^ is used and results are taken from [51].

A full study of burst localization in the first two years of the aLIGO-AdV network was completed in [51]. This study was conducted using cWB and LIB. Sky localization was quantified by the searched area. Using an approximate FAR threshold of 1 yr^−1^, the median localization was shown to be ∼ 100–200 deg^2^ for a two-detector network in 2015–2016 and ∼ 60–110 deg^2^ for a three-detector network in 2016–2017, with the localization and relative performance of the algorithms depending upon the waveform morphology. Results for Gaussian, sine-Gaussian, broadband white-noise and BBH waveforms are shown in Figure 7 (for the two-detector O1 network and the three-detector O2 network, cf. Figure 6). The largest difference between the codes here is for the white-noise bursts; for a three-detector network, LIB achieves better localization than cWB for Gaussian and sine-Gaussian bursts. The variety of waveform morphologies reflect the range of waveforms that could be detected in a burst search [16].

## Observing Scenario

In this section we estimate the sensitivity, possible number of detections, and localization capability for each of the observing runs laid out in Section 2.2. We discuss each future observing run in turn and also summarize the results in Table 1.

**Table 1:** Summary of a plausible observing schedule, expected sensitivities, and source localization with the advanced LIGO and Virgo detectors, which will be strongly dependent on the detectors’ commissioning progress. The burst ranges assume standard-candle emission of 10^−2^ *M*_⊙_*c*^2^ in gravitational waves at 150 Hz and scale as $E_{{\rm{GW}}}^{1/2}$, so it is greater for more energetic sources (such as binary black holes). The binary neutron-star (BNS) localization is characterized by the size of the 90% credible region (CR) and the searched area. For 2015–2016 and 2016–2017, these have been calculated from parameter-estimation studies (neglecting detector calibration uncertainty) [31, 99] using LALInference [110]. The CRs for subsequent periods are estimated from timing triangulation (highlighted by italics), which is known to provide estimates on average a factor of ∼ 4 too large for a three-detector network [60, 31], hence these serve as a conservative bound. Both ranges as well as the BNS timing-triangulation localizations reflect the uncertainty in the detector noise spectra shown in Figure 1. Differences in the shape of the detector noise curves and also relative sensitivities between detectors have an effect on the localization areas. The BNS detection numbers also account for the uncertainty in the BNS source rate density [13]. BNS detection numbers and localization estimates are computed assuming a signal-to-noise ratio greater than 12. Burst localizations are expected to be broadly similar to those derived from timing triangulation, but vary depending on the signal bandwidth; the median burst searched area (with a false alarm rate of ∼ 1 yr^−1^) may be a factor of ∼ 2–3 larger than the values quoted for BNS signals [51]. No burst detection numbers are given, since the source rates are currently unknown. Localization and detection numbers assume an 80% duty cycle for each instrument.

Epoch			2015–2016	2016–2017	2017–2018	2019+	2022+ (India)
Estimated run duration			4 months	6 months	9 months	(per year)	(per year)
Burst range/Mpc		LIGO	40–60	60–75	75–90	105	105
		Virgo	—	20–40	40–50	40–80	80
BNS range/Mpc		LIGO	40–80	80–120	120–170	200	200
		Virgo	—	20–60	60–85	65–115	130
Estimated BNS detections			0.0005–4	0.006–20	0.04–100	0.2–200	0.4–400
90% CR	% within	5 deg^2^	< 1	2	> *1–2*	> *3–8*	> *20*
		20 deg^2^	< 1	14	> *10*	> *8–30*	> *50*
	median/deg^2^		480	230	—	—	—
searched area	% within	5 deg^2^	6	20	—	—	—
		20 deg^2^	16	44	—	—	—
	median/deg^2^		88	29	—	—	—

In the following, we estimate the expected number of BNS coalescence detections using both the lower and upper estimates on the BNS source rate density, 10^−8^–10^−5^ Mpc^−3^ yr^−1^ [13]. Given the detectors’ noise spectral densities, the *ρ*_*c*_ detection threshold can be converted into the (source sky-location and orientation averaged) BNS sensitive detection range *R*_BNS_ [13, 20]. From this, the BNS source rate density can be converted into an estimate of the number of expected detected events; this estimate carries the large error on the source rate density. Similar estimates may be made for NS-BH binaries using the fact that the NS-BH range is approximately a factor of 1.6 larger than the BNS range,^5^ though the uncertainty in the NS-BH source rate density is slightly larger [13]. We assume a nominal *ρ*_*c*_ threshold of 12, at which the expected FAR is 10^−2^ yr^−1^. However, such a stringent threshold may not be appropriate for selecting candidate triggers for electromagnetic follow-up. For example, selecting CBC candidates at thresholds corresponding to a higher background rate of 1 yr^−1^ (100 yr^−1^) would increase the number of true signals subject to electromagnetic follow-up by about 30% (90%). The area localization for these low-threshold signals is only fractionally worse than for the high-threshold population — by approximately 20% (60%). The localization of NS-BH signals is expected to be similar to that of BNS signals.

For typical burst sources, the GW waveform is not well known. However, the performance of burst searches is largely independent of the detailed waveform morphology [14, 51], allowing us to quote an approximate sensitive range determined by the total energy *E*_GW_ emitted in GWs, the central frequency *f*_0_ of the burst, the detector noise spectrum *S*(*f*), and the single-detector SNR threshold *ρ*_det_ [101],

2 $${R_{{\rm{burst}}}} \simeq {\left[ {{G \over {2{\pi ^2}{c^3}}}{{{E_{{\rm{GW}}}}} \over {S({f_0})f_0^2\rho _{\det}^2}}} \right]^{1/2}}.$$

In this article, we quote ranges using *E*_GW_ = 10^−2^ *M*_⊙_*c*^2^ and *f*_0_ = 150 Hz; *E*_GW_ = 10^−2^ *M*_⊙_*c*^2^ is an optimistic value for GW emission from stellar collapse (see, e.g., [18]), the uncertainty in *E*_GW_ means that the quoted burst ranges are more uncertain than their BNS counterparts. We use a single-detector SNR threshold of 8, corresponding to a typical *ρ*_*c*_ ≃ 12 and FAR of ∼ 0.3 yr^−1^. Due to the tail of the low-frequency background-rate-vs-amplitude distribution in Figure 3, we see that varying the selection threshold from a background of 0.1 yr^−1^ (*ρ*_*c*_ ≳ 15) to even 3 yr^−1^ (*ρ*_*c*_ ≳ 10) would increase the number of true signals selected for electromagnetic follow-up by a factor (15/10)^3^ ∼ 3, though the area localization for low-SNR bursts may be particularly challenging.

The run durations discussed below are in calendar time. Based on prior experience, we can reasonably expect a duty cycle of ∼ 80% for each instrument during observing runs.^6^ Assuming downtime periods are uncorrelated among detectors, this means that all detectors in a three-detector network will be operating in coincidence approximately 50% of the time and two of the three detectors will be operating an additional 40% of the time. For a four-detector network, three or more detectors will be operational around 80% of the time. Our estimates for the expected number of detections and the fraction of sources localized account for these duty cycles. The number of detections also account for the uncertainty in the detector sensitive ranges as indicated in Figure 1.

### 2015–2016 run (O1): aLIGO 40–80 Mpc

This is the first advanced-detector observing run, lasting four months, starting 18 September 2015 and ending 12 January 2016. The aLIGO sensitivity is expected to be similar to the early band in Figure 1, with a BNS range of 40–80 Mpc, and a burst range of 40–60 Mpc for *E*_GW_ = 10^−2^ *M*_⊙_*c*^2^. Our experience so far indicates that sensitivity will probably be at the better end of this span, with a BNS range potentially in the interval 60–80 Mpc.

A four-month run gives a BNS search volume of (0.5–4) × 10^5^ Mpc^3^ yr at the confident detection threshold of *ρ*_*c*_ = 12. The search volume is (4/3)*πR*^3^ × *T*, where *R* is the range and *T* is the observing time incorporating the effects of the detectors’ duty cycles. We therefore expect 0.0005–4 BNS detections. A BNS detection is likely only if the most optimistic astrophysical rates hold.

With the two-detector H1-L1 network any detected events are unlikely to be well localized. A full parameter-estimation study using realistic detector noise and an astrophysically-motivated source catalog has been completed for 2015–2016 [31].^7^ This used a noise curve in the middle of the early range shown in Figure 1 (the early curve specified in [30]). The distribution of results is shown in Figure 6. It was found that using an SNR threshold of 12, the median 90% credible region for BNS signals is ∼ 500 deg^2^, and only 4% of events are expected to have ${\rm{CR}}_{0.9}^{{\rm{BNS}}}$ smaller than 100 deg^2^; the searched area $A_{\ast}^{{\rm{BNS}}}$ is smaller than 20 deg^2^ for 16% of events and smaller than 5 deg^2^ in 6%. If a FAR threshold of 10^−2^ yr^−1^ is used without the SNR cut, these localizations change because of the inclusion of an additional population of events with SNRs ∼ 10–12. The median ${\rm{CR}}_{0.9}^{{\rm{BNS}}}$ is ∼ 600 deg^2^ and 3% have ${\rm{CR}}_{0.9}^{{\rm{BNS}}}$ smaller than 100 deg^2^; $A_{\ast}^{{\rm{BNS}}}$ is smaller than 20 deg^2^ for 14% of events and smaller than 5 deg^2^ for 4%.

Equivalent (but not directly comparable) results for bursts are found in [51]. Specific results depend upon the waveform morphology used, but the median searched area is ∼ 1–2 times larger than for BNS signals; part of this difference is due to the burst study using a less-stringent FAR threshold of ∼ 1 yr^−1^. The distribution of searched areas for four waveform morphologies are shown in Figure 7.

Follow-up observations of a GW signal would be difficult, but not impossible. Localizations provided by another instrument, such as a gamma-ray burst telescope, could improve the possibility of locating an optical or a radio counterpart.

### 2016–2017 run (O2): aLIGO 80–120 Mpc, AdV 20–60 Mpc

This is envisioned to be a six-month run with three detectors. The aLIGO performance is expected to be similar to the mid band in Figure 1, with a BNS range of 80–120 Mpc, and a burst range of 60–75 Mpc for *E*_GW_ = 10^−2^ *M*_⊙_*c*^2^. The AdV range may be similar to the early band, approximately 20–60 Mpc for BNS and 20–40 Mpc for bursts. This gives a BNS search volume of (0.6–2) × 10^6^ Mpc^3^ yr, and an expected number of 0.006–20 BNS detections.

**Figure 8:**
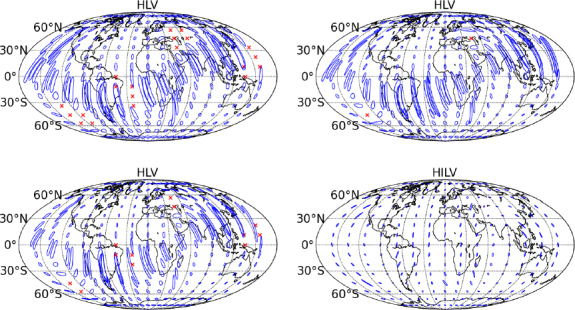
Schematic network sensitivity and localization accuracy for face-on binary neutron-star (BNS) systems with advanced-detector networks. The ellipses show 90% confidence localization areas based upon timing triangulation alone, and the red crosses show regions of the sky where the signal would not be confidently detected. The top two plots show the localization expected for a BNS system at 80 Mpc by the LIGO Hanford (H)-LIGO Livingston (L)-Virgo (V) network (HLV) in the 2016–2017 run (*left*) and 2017–2018 run (*right*). The bottom two plots show the localization expected for a BNS system at 160 Mpc by the HLV network in the 2019+ run (*left*) and by the four-detector network (HILV) comprising three LIGO sites — in Hanford, Livingston and India (I) — and Virgo operating in 2022+ with all detectors at final design sensitivity (*right*). The inclusion of a fourth site in India provides good localization over the whole sky.

Source localization for various points in the sky for CBC signals for the three-detector network is sketched in Figure 8.

BNS sky localization for 2016–2017 (in addition to 2015–2016) has been investigated in [99]. This assumed a noise curve which lies in the middle of the mid range in Figure 1 for aLIGO (the mid curve specified in [30]) and the geometric mean of the upper and lower bounds of the mid region in Figure 1 for AdV. The distribution of results is shown in Figure 6. Performing parameter estimation on an astrophysically-motivated BNS population, with an SNR threshold of 12 (in addition to a FAR cut of 10^−2^ yr^−1^), it was found that the median 90% credible region for BNS signals is ∼ 200 deg^2^, and 20% of events are expected to have ${\rm{CR}}_{0.9}^{{\rm{BNS}}}$ smaller than 20 deg^2^. The searched area is smaller than 20 deg^2^ for 44% of events and smaller than 5 deg^2^ for 20%. The burst study [51] gives approximately equivalent results, producing median searched areas a factor of ∼ 2–3 larger than the BNS results; these results are shown in Figure 7.

### 2017–2018 run (O3): aLIGO 120–170 Mpc, AdV 60–85 Mpc

This is envisioned to be a nine-month run with three detectors. The aLIGO and AdV sensitivities will be similar to the late and mid bands of Figure 1 respectively, with BNS ranges of 120–170 Mpc and 60–85 Mpc, and burst ranges of 75–90 Mpc and 40–50 Mpc for *E*_GW_ = 10^−2^ *M*_⊙_*c*^2^. This gives a BNS search volume of (3–10) × 10^6^ Mpc^3^ yr, and an expected 0.04–100 BNS detections. Source localization for CBC signals is illustrated in Figure 8. While the greater range compared to the 2016–2017 run increases the expected number of detections, the detector bandwidths are marginally smaller. This slightly degrades the localization capability for a source at a fixed SNR.

### 2019+ runs: aLIGO 200 Mpc, AdV 65–130 Mpc

At this point we anticipate extended runs with the detectors at or near design sensitivity. The aLIGO detectors are expected to have a sensitivity curve similar to the design curve of Figure 1. AdV may be operating similarly to the late band, eventually reaching the design sensitivity circa 2021. This gives a per-year BNS search volume of 2 × 10^7^ Mpc^3^ yr, giving an expected 0.2–200 confident BNS detections annually. Source localization for CBC signals is illustrated in Figure 8. The fraction of signals localized to areas of a few tens of square degrees is greatly increased compared to previous runs. This is due to the much larger detector bandwidths, particularly for AdV; see Figure 1.

### 2022+ runs: aLIGO (including India) 200 Mpc, AdV 130 Mpc

The four-site network incorporating LIGO-India at design sensitivity would have both improved sensitivity and better localization capabilities. The per-year BNS search volume increases to 4 × 10^7^ Mpc^3^ yr, giving an expected 0.4–400 BNS detections annually. Source localization is illustrated in Figure 8. The addition of a fourth detector site allows for good source localization over the whole sky [96, 109, 79, 91].

## Conclusions

We have presented a possible observing scenario for the Advanced LIGO and Advanced Virgo network of GW detectors, with emphasis on the expected sensitivities and sky-localization accuracies. This network began operations in September 2015. Unless the most optimistic astrophysical rates hold, two or more detectors with an average range of at least 100 Mpc and with a run of several months will be required for BNS detection.

Electromagnetic follow-up of GW candidates *may* help confirm GW candidates that would not be confidently identified from GW observations alone. However, such follow-ups would need to deal with large position uncertainties, with areas of many tens to thousands of square degrees. This is likely to remain the situation until late in the decade. Optimizing the electromagnetic follow-up and source identification is an outstanding research topic (see, e.g., [15, 5, 67, 99, 39, 52, 59]). Triggering of focused searches in GW data by electromagnetically-detected events can also help in recovering otherwise hidden GW signals.

Networks with at least two detectors with sensitivities of the order of 200 Mpc are expected to yield detections based purely on GW data after a few years of observation, even under the most pessimistic predictions of signal rates. Sky localization will continue to be poor until a third detector reaches a sensitivity within a factor of ∼ 2 of the others and with a broad frequency bandwidth. With a four-site detector network at final design sensitivity, we may expect a significant fraction of GW signals to be localized to within a few square degrees by GW observations alone.

The purpose of this article is to provide information to the astronomy community to facilitate planning for multi-messenger astronomy with advanced GW detectors. *While the scenarios described here are our best current projections, they will likely evolve as detector installation and commissioning progress*. We will therefore update this article regularly.

## Changes Between Versions

Since the arXiv-only first version [4], several updates to the document have been made. The key differences are outlined below.

### Updates to detector commissioning

The plausible detector scenarios remain largely unchanged, although the text has been updated to reflect our progress. With the aLIGO instruments now completed and both on a path of increasing sensitivity, and AdV advancing, the previous sensitivity evolution appears to remain on track. Modifications that have been made are:

1. While the O1 BNS range 40–80 Mpc has remained unchanged, our experience has led us to suggest a probable range within this interval of 60–80 Mpc; the range will not be fully determined until the completion of calibration for the run.
2. The length of O1 has been extended from three months to four months, taking the run into the early part of 2016. The search volume and expected number of detections have been revised proportionately.
3. The possibility of AdV joining aLIGO for O1 has been removed; this was always optimistic and none of the results assumed that this would be the case.
4. The potential time-line for LIGO-India has been pushed back. This will remain uncertain until funding is finalized, and forecasting progress on such long timescales is difficult.
5. The AdV 2019+ BNS range in Section 2.2 and Table 1 has been changed to 65–115 Mpc; this does not reflect a change in the timetable, but is just a clarification that a range of 130 Mpc is not expected until 2021.

A potential time-line for the sensitivity evolution and observing runs of detectors is sketched by Figure 2.

### Updates to sky localization

In addition to the progress made with regards to the detectors, there have also been advances in data analysis. The text has been updated to include discussions of the parameter-estimation codes that will be used in the upcoming observing runs; Section 3 has been reorganized to try to make this more straight-forward to understand. Specific results that have changed are:

1. The estimated localizations of the O1 (Section 4.1) and O2 (Section 4.2) have been updated to use results of full parameter-estimation studies. Results for these are shown in Figure 6 for BNSs and Figure 7 for bursts. Previously, no numbers were given for O1.
2. Table 1 has been updated to include the results of the O1 and O2 studies. Timing triangulation is still used to give an indication of sky localization for later observing runs, as full parameter-estimation studies have not yet been completed. These will be updated in the future, but currently serve to give rough (conservative) estimates [60, 31]. Parameter-estimation results for later observing runs will be included in the future.
3. Table 1 now also lists both the 90% credible region and the searched area; previously only the 90% area was listed.

An example BNS sky map, created using current parameter-estimation techniques, is shown in Figure 5.

## Footnotes

1 Another often quoted number is the BNS *horizon* — the distance at which an optimally oriented and located BNS system would be observed with an SNR of 8. The horizon is a factor of 2.26 larger than the range [58, 13, 20].

2 Data from the fifth and sixth science runs (S5 and S6) of LIGO are publicly available from the LIGO Open Science Center losc.ligo.org [108].

3 We do not intend to produce timing-only sky maps, but timing triangulation can be useful for order-of-magnitude estimates of sky-localization accuracy averaged across the population of signals.

4 We expect that on average 50% of sources should be found within a reported 50% confidence region, but this is not the case: between 65% and 85% of detected signals have been found within the 50% confidence regions, depending upon the detector network and signal morphology [51].

5 This assumes a black-hole mass of 5 *M*_⊙_.

6 This is consistent with what was achieved with the initial detectors, where H1 and L1 had duty cycles of 78% and 67% in the S5 observing run [21]. Virgo had duty cycles of 81%, 80% and 73% in observing runs VSR1–3 respectively [1].

7 This study used noise from the sixth science run of initial LIGO, recolored to the expected O1 sensitivity curve. The source catalog, as well as the analysis pipeline, is shared with [99].
